# Towards Development of a 3-State Self-Paced Brain-Computer Interface

**DOI:** 10.1155/2007/84386

**Published:** 2007-10-10

**Authors:** Ali Bashashati, Rabab K. Ward, Gary E. Birch

**Affiliations:** ^1^Electrical and Computer Engineering Department, University of British Columbia, Vancouver, BC, Canada V6T 1Z4; ^2^Terry Fox Laboratory, British Columbia Cancer Research Center, Vancouver, BC, Canada V5Z 1L3; ^3^Brain Interface Laboratory, Neil Squire Society, Suite 220, 2250 Boundary Road, Burnaby, BC, Canada V5M 3Z3; ^4^Institute for Computing, Information & Cognitive Systems, Vancouver, BC, Canada V6T 1Z4

## Abstract

Most existing brain-computer interfaces (BCIs) detect specific mental activity in a so-called synchronous paradigm. Unlike synchronous systems which are operational at specific system-defined periods, self-paced (asynchronous) interfaces have the advantage of being operational at all times. The low-frequency asynchronous switch design (LF-ASD) is a 2-state self-paced BCI that detects the presence of a specific finger movement in the ongoing EEG. Recent evaluations of the 2-state LF-ASD show an average true positive rate of 41% at the fixed false positive rate of 1%. This paper proposes two designs for a 3-state self-paced BCI that is capable of handling idle brain state. The two proposed designs aim at detecting right- and left-hand extensions from the ongoing EEG. They are formed of two consecutive detectors. The first detects the presence of a right- or a left-hand movement and the second classifies the detected movement as a right or a left one. In an offline analysis of the EEG data collected from four able-bodied individuals, the 3-state brain-computer interface shows a comparable performance with a 2-state system and significant performance improvement if used as a 2-state BCI, that is, in detecting the presence of a right- or a left-hand movement (regardless of the type of movement). It has an average true positive rate of 37.5% and 42.8% (at false positives rate of 1%) in detecting right- and left-hand extensions, respectively, in the context of a 3-state self-paced BCI and average detection rate of 58.1% (at false positive rate of 1%) in the context of a 2-state self-paced BCI.

## 1. INTRODUCTION

Brain-computer interface (BCI) systems form a possible alternative communication and control solutions for individuals with severe disabilities. For a review of the field, see [1–3]. In BCI systems, the user's cortical activity associated with an intentional control of a device (such as attempted finger movements) is directly mapped to an application-specific control signal. This allows the user to control various devices such as a neural prosthetic by cognitive processes only, that is, by bypassing traditional interface pathways (which cannot be used by individuals with severe disabilities).

In developing noninvasive BCI systems, the majority of research has concentrated on developing synchronous systems. These systems are only operational at specific periods. Asynchronous (self-paced) systems, on the other hand, have the advantage of being operational at all times. The 2-state low frequency-asynchronous switch design (LF-ASD) was the first BCI introduced for self-paced or asynchronous control applications [4]. LF-ASD seeks to recognize the movement related potentials (MRPs) of a *finger-flexion movement* in the EEG signal. In a self-paced brain-computer interface the users affect the BCI transducer output whenever they want by intentionally changing their brain states. Between periods of intentional control (IC), users are said to be in a no-control (NC) state; they may be idle, daydreaming, thinking about a problem or lunch, or performing any other action other than trying to control the BCI transducer. These BCI transducers are thus designed to respond only when there is an intentional user control. The appropriate BCI response to no-control (NC) would be a neutral or inactive output. We refer to this ability as NC support. NC support is necessary for most types of machine or device interactions where frequent intentional controls (IC) are spaced by periods of inaction.

Like LF-ASD, the 2-state BCI systems tested in [5–7] attempt to detect an intentional control state from the ongoing brain signal in a self-paced manner. The 3-state self-paced BCI implemented in [8] attempts to differentiate between right-hand, left-hand, and foot movements to operate a virtual keyboard. However, this BCI requires the subject to constantly engage in control without the option of going to the no control (NC) state. In a recent work, Scherer et al. [9] has proposed a 4-state self-paced BCI that has mean true positive and false positive rates of 28.4% and 16.9%. In the study of [10] the subjects were asked to perform one of the following three actions: (1) imagine right-hand movement, (2) imagine left-hand movement, and (3) relax. A 3-state self-paced BCI was designed to navigate a mobile robot in an 80 cm∗60 cm house-like environment by differentiating amongst these three states. The system generates “unknown state output” when there is not enough confidence in choosing one of the three above-mentioned mental tasks. The classifier of this system was not explicitly trained to recognize idle (NC) state [10]. According to the authors, it could process them adequately by responding “unknown”. It was also reported that the task of steering the robot between rooms was so engaging that the two tested subjects preferred to emit continuously mental commands rather than to go through idle state. Therefore, the response of this system on NC (idle) state was evaluated on a dataset with limited amount of idle-state. Moreover, having the choice of “unknown state output” may represent some neutral output but it is not clear whether the unknown state output was caused by the actual idle (NC) state or by lack of confidence in detecting one of the three commands. Additionally, there is no evidence that the NC state will fall into the unknown state in these designs.

In this paper, a noninvasive 3-state self-paced BCI system is proposed. This system is a 3-state self-paced BCI that is (a) designed specifically to support the NC state EEG signal, and (b) has a higher true positive rate at a considerably lower false positive rate (FP=1%) compared to existing 3-state and 4-state self-paced BCIs that support the NC state [9]. It should, however, be mentioned that it is difficult to directly compare the results of our study with other BCI studies because (a) the recording equipment, recording and classification protocols, and mental tasks considered are different, (b) the amount of data involved and the degree of training the subjects received before and during participation in the BCI experiments varies for different studies, and (c) there is not a unified framework of reporting performance of BCI systems, that is, the performance metrics are different across different studies.

**Figure 1:**
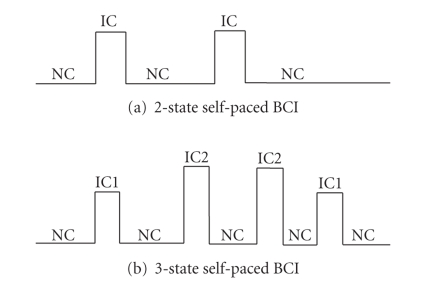
Samples of outputs of 2-state and 3-state self-paced BCIs, where NC = no-control state, IC = intended control state.

Unlike the 2-state self-paced system which detects the presence of a single movement from the ongoing EEG signal, the 3-state self-paced BCI design aims at detecting two different movements. Figure 1 shows examples of outputs of the 2-state and 3-state self-paced BCIs. Overall, a 2-state self-paced BCI is in an inactive state (NC state) for most of the time and is in an IC state when a specific brain state (e.g., finger-flexion movement) is detected in the brain signal. Unlike a 2-state self-paced BCI which has only one active (IC) state, a 3-state self-paced BCI has two active state outputs, IC1 and IC2, which are activated by two different brain states (e.g., right- and left-hand extensions). While a 2-state self-paced BCI can provide the user with the option of executing only one command (e.g., turn right), a 3-state system gives the user two command options (e.g., turn right or turn left). This has the advantage of giving the user more control options.

The 2-state self-paced BCI (LF-ASD) in [4] aimed at detecting attempted right finger flexions. Recent studies with the 2-state LF-ASD have demonstrated that this system correctly detects the presence of a movement (true positive (TP) rate) in 41% and 42% of the cases for able-bodied and spinal-cord-injured subjects, respectively [11]. This is when the parameters were set so that the false positive rate is fixed at 1%. The TP rate of the system improves at higher FP rates, for example, at an FP rate of 5%, the TP rate is almost 100%. Despite these encouraging results, our experience indicates that even a 1% false positive rate is too high for most practical self-paced control applications.

This paper reports on the preliminary results of a pilot study that investigates the feasibility of a 3-state “self-paced” brain-computer interface system whose aim is the detection of right- and left-hand extension movements in a self-paced manner. This system has the ability to handle the no-control (NC) state as well as two additional control options for the user.

Two consecutive detectors were designed to detect the presence of the left- or the right-hand extensions from the ongoing EEG. The first detector, DET1, determines whether or not a movement is present. If such a movement is detected then the second detector, DET2, classifies the movement as a right- or a left-hand extension.

Two designs of a 3-state self-paced BCI are proposed and implemented. Power spectral density and a specific template matching method [4] are used in the feature extraction stages, and the k-nearest neighbour and linear discriminant analysis (LDA) classifiers are used in the classification stages.

The performances of the designs are evaluated using EEG recordings of right- and left-hand extension movements of four able-bodied individuals. The goals of this paper are twofold.

1. To perform an initial investigation of the performance of the system as a 2-state self-paced BCI, that is, detecting whether a left- or a right-hand movement (regardless of the type of movement) has occurred. If the performance of the system in detecting any such movement is better than detecting the previously used movement (i.e., the right-finger flexion), then such these movements can be used in other 2-state self-paced brain-computer interface designs.
2. To introduce and carry out an initial evaluation of two possible designs of a 3-state self-paced BCI and to investigate whether a 3-state self-paced brain-computer interface that supports the no-control (NC) state has promise.

In Sections 2–4 of this paper, details of experiments, the structure of the proposed designs and the evaluation method are explained. The results, discussions, and conclusions are presented in Sections 5–7, respectively.

## 2. EXPERIMENTS

### 2.1. Selection of movement tasks

A 3-state self-paced BCI has two active state outputs, IC1 and IC2, which should be activated by two different movements (as neurophysiologic sources of control). However, selection of these movements is not a trivial task and one needs to find the movements that generate more differentiable patterns in the EEG. More differentiable patterns would make it easier for a BCI system to detect IC states and may yield improvements in the performance of the system.

Many studies by the neurophysiologic research community have explored the effects of different movements on the EEG signal. These studies show that movements that involve more parts of the body (e.g., hand movement) or movements that need more effort (e.g., finger extension) generate more differentiable patterns in the ongoing EEG signal than for example natural finger flexions [12–14]. It has also been reported that right and left movements (regardless of the type of movement) generate patterns in different locations of the brain [15]. As our aim is to use movements that generate more differentiable patterns, based on the evidence in [12–15], we choose the right-hand and the left-hand extensions in this study since (a) hand movements involve more parts of the body than, for example, finger movements, (b) extension movements need more effort to execute compared to flexion movements, and (c) right and left movements generate movement-specific patterns in different locations of the brain. We speculate that these two movements generate more discriminative patterns than a finger flexion does. If that is the case, then using these movements would improve our BCI's performance in detecting the presence of a movement. To our best knowledge, the right- and the left-hand extension movements have not yet been studied in the context of BCI systems.

### 2.2. Experimental paradigm

The EEG data used in this study were recorded from 15 monopolar electrodes positioned over the supplementary motor area and the primary motor cortex (defined with reference to the International 10–20 System at F1, F2, F3, F4, Fz, FC1, FC2, FC3, FC4, FCz, C1, C2, C3, C4, and Cz). Electro-oculographic (EOG) activity was measured as the potential difference between two electrodes, placed at the corner and below the right eye. The ocular artifact was considered present when the difference between the EOG electrodes exceeded +/−25 *μ*v, a threshold level similar to the one used in previous studies [3, 15]. All signals were sampled at 128 Hz. This study has been approved by the Behavioural Research Ethics Board (BREB) of the University of British Columbia.

**Figure 2:**
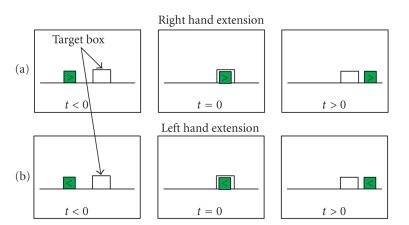
Screen contents for each of the right-hand (a) and left-hand (b) extension movement trials, t=0 is the time of movement execution.

Four able-bodied subjects participated in this study. All subjects were male (except subject 4), right-handed (except subject 4), 25–30 years old, and only subject 2 had prior BCI experience. Subjects were seated 150 cm in front of a computer monitor. The data were collected while the subjects were performing a cue-based (synchronized) task. At random intervals of 5.6–7 seconds (mean of 6.7 seconds), a target window was displayed on the subject's monitor. As shown in Figure 2, a box moved from the left side to the right side of the screen. When the box reached the target window, the subject attempted to activate the custom-made switch by extending his/her right- or left-hand. An arrow in the moving box, pointing to the left or the right showed the subject whether to move the right- or the left-hand. For each subject, an average of 150 trials for each movement was collected in two sessions carried in the same day.

## 3. PROPOSED 3-STATE SELF-PACED BRAIN COMPUTER INTERFACE

**Figure 3:**
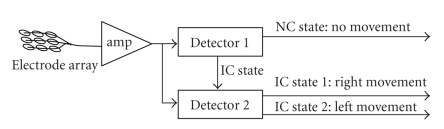
Structure of the 3-state self-paced brain-computer interface design.

Figure 3 shows the overall structure of the proposed designs. These designs include two major blocks:

- “Detector 1” which determines whether or not a movement is performed, and
- “Detector 2” which determines whether the detected movement is a right-hand or a left-hand extension.

In this study, two different designs for Detector 1 and one design for Detector 2 have been proposed and evaluated. The details of both detectors are explained below. Detectors 1 and 2 are referred to as DET1 and DET2.

### 3.1. Detector 1

Two different designs for DET1 are proposed and compared. These are referred to as DET1-LF-1NN and DET1-PSD-LDA.

**Figure 4:**
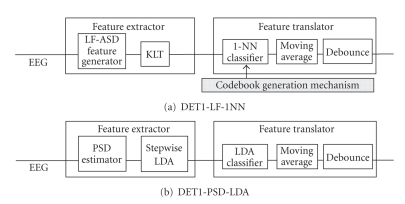
Structure of the two designs of DET1, where KLT = Karhunen-Loève transform, and 1-NN = 1-nearest neighbour, PSD = power spectral density, and LDA: linear discriminant analysis.

DET1-LF-NN uses one of the latest designs of the LF-ASD [14] as shown in Figure 4(a). It employs features extracted from the 0–4 Hz band in six bipolar EEG channels (defined with reference to the International 10–20 System at F1-FC1, Fz-FCz, F2-FC2, FC1-C1, FCz-Cz, and FC2-C2). After amplification, a lowpass FIR filter (0–4 Hz) is used to decrease the interference with the features in the high-frequency band.

**Figure 5:**
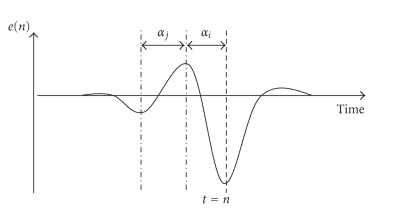
Description of delay terms (*α*_*i*_, *α*_*j*_), where e(n) is the amplitude of the bipolar signal.

Previous studies show that when a movement is performed, a bipolar pattern similar to the one shown in Figure 5 is generated in the ongoing EEG [4]. A specific template matching algorithm based on the one employed in [4] is implemented. This algorithm generates large feature values when there is such a pattern in the spontaneous EEG. The delay parameters *α*_*i*_ and *α*_*j*_, shown in Figure 5, determine the locations of the peaks of the pattern that need to be detected. Thus, these delay parameters need to be properly determined in order to detect the presence of a specific movement. For each subject, the ensemble averages of the EEG around the movements of the training data are generated and then used to determine the values of *α*_*i*_ and *α*_*j*_ according to the method presented in [16]. Table 1 shows the mean values of *α*_*i*_ and *α*_*j*_ across all five runs (refer to Section 4) that are estimated from the ensemble averages of the training data of each run. This feature extraction procedure is repeated for each of the six bipolar channels. The resulting feature vector is a six-dimensional vector, with each dimension reflecting the value of the feature in each channel. While we used the same *α*_i_ and *α*_j_ parameter values for all the six channels because the evidence in [16] suggests that they are not significantly different, we have also checked the ensemble averages of all the six channels to make sure that this assumption is valid in this study.

The Karhunen-Loève transform (KLT) component is used to reduce the 6-dimensional feature vector to a 2-dimensional feature vector. A 1-NN (1-nearest neighbour) classifier is used as the feature classifier. Finally, a moving average (with length of 39 milliseconds) and a debounce block (with length of 125 milliseconds) are employed to further improve the classification accuracy of DET1 by reducing the number of false activations (for details, see [4, 17]). DET1 classifies the input patterns, at every 1/16th of a second, to one of the two classes, no-control (NC) or intentional-control (IC) states.

The second design of DET1 (referred to as DET1-PSD-LDA) is shown in Figure 4(b). It extracts the power spectral density features of the EEG from a group of bipolar EEG channels and then selects the most informative channels for classification. Specifically, thirty bipolar combinations of EEG channels that may contribute to the detection of movements were generated. These bipolar EEG channels were Cz-C1, Cz-C2, Cz-C3, Cz-C4, C1-C2, C1-C4, C1-C3, C2-C3, C2-C4, C3-C4, FCz-Cz, FC1-C1, FC2-C2, FC3-C3, FC4-C4, Fz-FCz, F1-FC1, F2-FC2, F3-FC3, F4-FC4, FCz-FC1, FCz-FC2, FCz-FC3, FCz-FC4, FC1-FC2, FC1-FC4, FC1-FC3, FC2-FC3, FC2-FC4, FC3-FC4. These bipolar channels were chosen to capture possible discriminatory information between left and right and also between frontal and central areas of the head. In the feature extraction block, the power spectral density (PSD) components of each of the 30 bipolar EEG channels are calculated in each frequency bin from 1 Hz to 25 Hz using Welch's Periodogram method [18] with window length of one second (equivalent to 128 samples). This results in 25 frequency components for each of the 30 bipolar channels and a total of 25∗30 features at each time instant. We then use stepwise linear discriminant analysis (stepwise LDA) [19] to find the most informative features that better discriminant between IC and NC classes. Stepwise LDA is a method that results in a linear combination of selected features that contribute to the classification and omits the features that have redundant information for discrimination. Once the features are extracted and selected, a linear discriminant classifier (LDA) [19] is used for classification. Other details about the other components of the feature translator (moving average and debounce blocks) are the same as in DET1-LF-1NN above.

### 3.2. Detector 2

Existing studies show that the cortical activation, related to movement preparation and execution, desynchronizes the alpha (8–12 Hz) rhythm and increases the beta (13–25 Hz) rhythm of the EEG. These phenomena are known as event-related desynchronization (ERD) and event-related synchronization (ERS), respectively [15, 20]. The ERD of a hand movement is more prominent over contralateral sensorimotor areas during motor preparation and extends bilaterally after movement initiation [15, 21]. Some studies, however, show that the frequency bands of the ERD and ERS patterns are variable from subject to subject [22].

**Figure 6:**
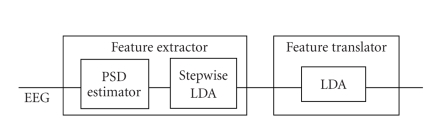
Structure of DET2-PSD-LDA, where PSD = power spectral density, and LDA = linear discriminant analysis.

As shown in Figure 6, DET2 which aims at differentiating between right- and left-hand movements is similar to the second design of DET1 (DET1-PSD-LDA), except that it does not have the averaging and debounce blocks of DET1. This design intends to extract subject specific ERD/ERS frequency bands that lead to more discrimination between the two classes, that is, the left- and right-hand movements. As in DET1, the stepwise linear discriminant analysis (LDA) method is employed to select the subject specific ERD/ERS frequency bands and bipolar EEG channels. We have evaluated a similar design of DET2 when the inputs were monopolar EEG channels. Preliminary analysis of the data shows that using bipolar electrodes yields better performances. As such, we used bipolar electrodes as input to the system and did not further evaluate the overall performance of the 3-state brain-computer interface using monopolar electrodes.

Two designs of a 3-state self-paced BCI system are evaluated. The first design uses the combination of DET1-LF-1NN and DET2-PSD-LDA and the second one uses the combination of DET1-PSD-LDA followed by DET2-PSD-LDA.

## 4. EVALUATION

The designed 3-state self-paced BCI first detects whether or not a movement is performed. If a movement is detected, then the system classifies it as one of two classes, the right-hand (IC1) or the left-hand (IC2) extension classes. If the system does not detect a movement, the output reports an inactive state.

We use 80% randomly chosen trials (about 120 trials) to train the 3-state self-paced BCI system and use the remaining data to evaluate the performance of the system. We repeat this procedure five times and report the mean performance of the system. The ability of the subjects to control the 3-state BCI system is evaluated using three performance measures. At a fixed false positive rate, these measures report the correct detection rates of the right- and the left-hand extensions (from the ongoing EEG), respectively. These three measures are as follows.

1. The percentage of correct right-hand movement detection during IC states (i.e., the true positive rate for right-hand movement, ${\text{TP}}_{\text{R}}$) calculated using (1) below:
  (1) $${\text{TP}}_{\text{R}}=\frac{\text{number of correctly detected right movements}}{\text{total number of right movements}}.$$
2. The percentage of correct left-hand movement detection during IC states (true positives of left-hand movements, ${\text{TP}}_{\text{L}}$) calculated using (2) below:
  (2) $${\text{TP}}_{\text{L}}=\frac{\text{number of correctly detected left movements}}{\text{total number of left movements}}.$$
3. The percentage of false switch activations during NC states (false positives, FPs) calculated using (3) below:
  (3) $$\text{FP}\;=\;\frac{\text{number of false activations}}{\text{total number of the system's decisions during NC state}}.$$

Note that the system make a decision every 1/16th of a second.

A TP is identified if the BCI system is activated at least once in a response window, that is, a time window spanning 0.25 seconds before the time of movement till 0.5 seconds after it, a method similar to that employed in [4, 5, 7, 23, 24]. FPs are assessed in the periods outside the response window as explained above. Periods during which ocular artifacts occurred are blocked from analysis.

We also report the overall true positive and false positive rates of DET1 (regardless of the type of movement). We refer to these measures as ${\text{TP}}_{\text{IC}}$ and ${\text{FP}}_{\text{IC}}$. The ${\text{TP}}_{\text{IC}}$ is the percentage of correct detection of a movement whether it is a right-hand or a left-hand one. Thus it reflects the performance of the system if used as a 2-state self-paced BCI. We report this measure to compare the findings of this study with our latest 2-state self-paced BCI as stated in goal (1) of this study.

## 5. RESULTS

The mean performance of DET1 (${\text{TP}}_{\text{IC}}$) in detecting the presence of hand movements, regardless of the type of movement, from the background EEG is shown in Table 2. This table shows the TP rates at a fixed FP rate of 1% for the two designs of DET1. As we are interested in low false positive rates, we do not report the performance of the system for higher false positive rates. For higher false positive rates (e.g., FP > 3%) the true positive rate is almost 100%. As shown in the last column of Table 2, the mean performance of DET1-LF-1NN is slightly better than DET1-PSD-LDA. For subject 2, the mean true positive rate of DET1-PSD-LDA is more than 15% higher than that of DET1-PSD-1NN with significance level of P < .03 using “paired t-test”. For subject 3, however, the differences between the performances of DET1-PSD-LDA and DET1-PSD-1NN are not significant at the significance level of 0.05. In the rest of the two subjects, the mean true positive rates of DET1-LF-1NN outperform DET1-PSD-LDA by more than 10% with significance levels of P < .02 through the use of “paired t-test”.

Table 3 shows the mean performance of the whole 3-state self-paced BCI for the two proposed designs (i.e., <DET1-LF-1NN + DET2-PSD-LDA> and <DET1-PSD-LDA + DET2-PSD-LDA>) at a fixed false positive rate of 1%.

On average, 36% of the right- and left-hand extensions of the 4 subjects are correctly identified by the 3-state <DET1-LF-1NN + DET2-PSD-LDA> design (for a false positive rate of 1%). As shown in Table 3, <DET1-LF-1NN + DET2-PSD-LDA> outperforms <DET1-PSD-LDA + DET2-PSD-LDA> in three of the tested subjects.^1^

Table 4 shows the best performing 3-state self-paced BCI design for each individual subject. As the last column of Table 4 shows, the average performance of the 3-state system achieves an overall true positive rate of 40.1% (at false positive rate of 1%). If used as a 2-state BCI its average true positive is 58.1%.

## 6. DISCUSSION

The proposed 3-state self-paced BCI was specifically designed to support NC state. This system was tested in a specific experimental paradigm and on NC state data that were supposed to be the most difficult one as they were surrounded by IC state data. However, a more thorough study is needed to investigate the performance of the system under different experimental paradigms and on different sets of NC state data, for example, when the person perform different mental tasks except for the IC task. This study would provide a better estimate of the performance of a self-paced BCI system in a real-world application.

The performance of DET1-LF-1NN and DET1-PSD-LDA in detecting the presence of a movement (regardless of its type) yielded average true positive rates of 54% and 53.4% at false positive (FP) rate of 1%, respectively. In the meantime, as shown in the third column of Table 4, the average ${\text{TP}}_{\text{IC}}$ rate for the best performing design across the subjects was 58.1% at false positive rate of 1%. In other words, if the current system was used as a 2-state self-paced BCI, the true positive rate would be 58.1% at false positive of 1%. In comparison, the results of the latest 2-state self-paced BCI [11] for four able-bodied subjects yielded an average true positive rate of 41% at the same false positive rate of 1%. Thus, when used as a 2-state system the proposed BCI performs significantly better than the 2-state self-paced BCI system in [11]. It should be noted that while this 2-state self-paced brain computer interface detects finger flexions [11], DET1 of the 3-state self-paced BCI detects the presence of a left- or a right-hand extension movement. This improvement should be the result of using hand extension movements instead of a finger flexion one. It should be noted however that direct comparison of the current system with [11] is not completely accurate as the data and experimental paradigms used in testing the two systems were different; a more thorough study is needed to verify these findings. Verifying these results on a very large subject pool would eventually provide a better neurophysiological source for controlling current 2-state self-paced BCIs.

As shown in Table 2, the overall performance of the 3-state BCI varies across the subjects and depends on the type of the design used. Such performance variability across different designs and subjects has also been observed in other BCI systems (e.g., [24, 25]). Given the variable performance of subjects across the two designs, an approach that can select a suitable design and adapt to each subject is expected to achieve better detection rates. Significant gains may also be achieved from the combination of several single designs if these designs provide complementary information for the classification task. Several studies have demonstrated some evidence of existing independent features related to movement tasks that could be used to achieve better classification accuracies [26–28].

Subject 4 yielded the best right and left true positive rates (${\text{TP}}_{\text{R}}$ and ${\text{TP}}_{\text{L}}$) of 53.3% and 54.7% at false positive rate of 1%, respectively. Although DET1's true positive rate in detecting the presence of a movement (${\text{TP}}_{\text{IC}}$) for subject 3 was the second best, overall the system has poor performance in differentiation between right and left movements. The following reasons might have caused the poor performance related to this subject.

- This subject did not generate significantly differentiable ERD/ERS patterns for the left- and right-hand movements. Many factors such as task complexity, effort and attention during the task can also contribute to the quality of the ERD/ERS patterns [15]. Other studies such as [29] have reported some subjects who poorly performed (classification rates of close to chance) compared to the rest of the subjects.
- In the experimental paradigm used in this study, no feedback during the performed tasks was provided to the subjects. While we adopted this paradigm to simulate a more natural mode of control, this may have caused a lower performance in some subjects.
- No subject prescreening and prior training was performed before the sessions.

Previous findings [17, 30] show that spinal-cord-injured (SCI) subjects can operate a self-paced BCI with almost the same results as able-bodied subjects. Thus, able-bodied subjects using a real movement are good predictors of the controllability of our proposed BCI system by SCI subjects using an attempted movement. It should be noted, however, that the findings of this study should be confirmed on our target population (i.e., individuals with motor disabilities) in future studies.

## 7. CONCLUSION

This study introduced and evaluated two designs of a 3-state self-paced brain-computer interface based on movement related potentials. This 3-state self-paced brain-computer interface is the first of its kind in its capability in (1) supporting the NC state, and (2) generating low false positive rates. While the true positive rate of the latest 2-state self-paced BCI is 41% (at FP = 1%) [11], the best average true positive rate of the proposed 3-state system is 40.1% (at FP = 1%). These results show that the 3-state system performs almost the same as the latest 2-state self-paced BCI [11] with the advantage of providing more control options than a 2-state system.

This preliminary study was performed to examine the feasibility of a 3-state “self-paced” brain-computer interface design. Although the results are promising, more improvements are needed in both of its components, that for detecting a movement and that for differentiating between two movements. The true positive rate of the system is reported at a false positive rate of 1%. Even a false positive rate of 1% is still not suitable for real-world applications as it corresponds to an average of one false activation every six seconds and may cause excessive user frustration. Use of more efficient feature extraction and classification methods, subject training, providing online feedback during the performed task, and verifying the results on a large number of subjects are in the scope of our future directions to improve these results.

## Figures and Tables

**Table 1:** Estimated mean values of *α*_*i*_ and *α*_*j*_ parameters for each subject. Note all values are in milliseconds.

	Subject 1	Subject 2	Subject 3	Subject 4
*α*_*i*_	125	195	398	195
*α*_*j*_	578	141	297	313

**Table 2:** Mean percentages of true positives (${\text{TP}}_{\text{IC}}$) at fixed false positive rate of 1% for the two designs of DET1.

DET1 Design	Subject 1	Subject 2	Subject 3	Subject 4	Average
DET1-LF-1NN	50.1	38.4	56.5	71.0	54.0
DET1-PSD-LDA	38.2	54.7	60.2	60.3	53.4

**Table 3:** Mean percentages of right and left true positives (${\text{TP}}_{\text{R}}$ and ${\text{TP}}_{\text{L}}$) of the two proposed 3-state brain-computer interfaces (when false positive rate is set at 1%). The ${\text{TP}}_{\text{R}}$ and ${\text{TP}}_{\text{L}}$ value of the best design combination for each subject is highlighted.

3-state BCI Design structure	Subject 1		Subject 2		Subject 3		Subject 4		Average
	${\text{TP}}_{\text{R}}$	${\text{TP}}_{\text{L}}$	${\text{TP}}_{\text{R}}$	${\text{TP}}_{\text{L}}$	${\text{TP}}_{\text{R}}$	${\text{TP}}_{\text{L}}$	${\text{TP}}_{\text{R}}$	${\text{TP}}_{\text{L}}$	
<DET1-LF-1NN + DET2-PSD-LDA>^1^	**30.6**	**32.6**	16.1	33.4	**30.5**	**36.7**	**53.3**	**54.7**	36.0
<DET1-PSD-LDA + DET2-PSD-LDA>	19.5	22.2	**35.6**	**47.0**	30.1	34.3	37.4	45.2	33.9

**Table 4:** Best design combination for each subject together with the performances of the 2-state and 3-state systems (at false positive of 1%), where A = <DET1-LF-1NN + DET2-PSD-LDA> and B = <DET1-PSD-LDA + DET2-PSD-LDA>.

Subject	Best design	2-state BCI	3-state BCI		
		${\text{TP}}_{\text{IC}}$	${\text{TP}}_{\text{R}}$	${\text{TP}}_{\text{L}}$	Average TP (${\text{TP}}_{3\text{-}\text{state}}$)
Subject 1	A	50.1	30.6	32.6	31.6
Subject 2	B	54.7	35.6	47	41.3
Subject 3	A	60.2	30.5	36.7	33.6
Subject 4	A	71.0	53.3	54.7	54
Average	—	58.1	37.5	42.8	40.1

## Footnotes

^1^Note that <DET1-LF-1NN + DET2-PSD-LDA> indicates a design of a 3-state BCI that uses DET1-LF-1NN design for DET1 and DET2-PSD-LDA design for DET2. A similar description applies to <DET1-PSD-1NN + DET2-PSD-LDA> design as well.
